# Warfarin Treatment Is Associated to Increased Internal Carotid Artery Calcification

**DOI:** 10.3389/fneur.2021.696244

**Published:** 2021-07-12

**Authors:** Krista Nuotio, Suvi M. Koskinen, Laura Mäkitie, Jarno Tuimala, Petra Ijäs, Hanna M. Heikkilä, Jani Saksi, Pirkka Vikatmaa, Pia Sorto, Sonja Kasari, Ilari Paakkari, Heli Silvennoinen, Leena Valanne, Mikko I. Mäyränpää, Lauri Soinne, Petri T. Kovanen, Perttu J. Lindsberg

**Affiliations:** ^1^Neurology, Neurocenter, Helsinki University Hospital, Helsinki, Finland; ^2^Clinical Neurosciences, Clinicum, University of Helsinki, Helsinki, Finland; ^3^Medical Imaging Center, Radiology, University of Helsinki and Helsinki University Hospital, Helsinki, Finland; ^4^Independent Researcher, Helsinki, Finland; ^5^Abdominal Center, Vascular Surgery, Helsinki University Hospital, Helsinki, Finland; ^6^Pharmacology, Faculty of Medicine, University of Helsinki, Helsinki, Finland; ^7^Pathology, Helsinki University and Helsinki University Hospital, Helsinki, Finland; ^8^Wihuri Research Institute, Biomedicum Helsinki 1, Helsinki, Finland

**Keywords:** carotid artery, warfarin, computed tomography angiography, histology, vascular calcification, calcification

## Abstract

**Background:** Long-term treatment with the vitamin K antagonist warfarin is widely used for the prevention of venous thrombosis and thromboembolism. However, vitamin K antagonists may promote arterial calcification, a phenomenon that has been previously studied in coronary and peripheral arteries, but not in extracranial carotid arteries. In this observational cohort study, we investigated whether warfarin treatment is associated with calcification of atherosclerotic carotid arteries.

**Methods:** Overall, 500 consecutive patients underwent carotid endarterectomy, 82 of whom had received long-term warfarin therapy. The extent of calcification was assessed with preoperative computed tomography angiography, and both macroscopic morphological grading and microscopic histological examination of each excised carotid plaque were performed after carotid endarterectomy.

**Results:** Compared with non-users, warfarin users had significantly more computed tomography angiography-detectable vascular calcification in the common carotid arteries (odds ratio 2.64, 95% confidence interval 1.51–4.63, *P* < 0.001) and even more calcification in the internal carotid arteries near the bifurcation (odds ratio 18.27, 95% confidence interval 2.53–2323, *P* < 0.001). Histological analysis revealed that the intramural calcified area in plaques from warfarin users was significantly larger than in plaques from non-users (95% confidence interval 3.36–13.56, *P* = 0.0018).

**Conclusions:** Long-lasting warfarin anticoagulation associated with increased calcification of carotid atherosclerotic plaques, particularly in locations known to be the predilection sites of stroke-causing plaques. The clinical significance of this novel finding warrants further investigations.

## Introduction

An atherosclerotic lesion in the internal carotid artery is a major cause of cerebral ischemic stroke. Although many elements of the underlying pathological processes of atherosclerosis, e.g., lipid accumulation and the inflammatory component, have been well-characterized in developing atherosclerotic lesions (1), the multifaceted roles of calcification in atherosclerotic lesions are still debated and under investigation (2–5).

Atrial fibrillation (AF), the most common sustained arrhythmia (6) poses a significant risk for cerebral embolism, which is most effectively prevented by anticoagulants (7–9). Both warfarin and modern oral anticoagulants are available and neurologists are frequently deciding on anticoagulation on patients with AF, often with simultaneous large artery atherosclerosis.

Warfarin has been claimed to have harmful effects on the arterial wall. Evidence from experimental animals has demonstrated that treatment with warfarin is linked to vascular calcification (10, 11), with similar findings from preliminary human studies (12–14). Human studies have suggested that exposure to warfarin may increase calcification in coronary arteries (15, 16), peripheral arteries (17), aorta (18), and aortic valve leaflets (19).

However, there are only a few studies that have investigated the association of warfarin and vascular calcification in carotid arteries (20, 21), and none of them has studied vascular calcification in extracranial carotid arteries. Hence, the present clinical investigation was undertaken to evaluate the hypothesis that chronic warfarin use is associated with vascular calcification in atherosclerotic carotid artery disease. We examined the preoperative computed tomography angiography (CTA) results, macroscopic calcification of the dissected carotid specimens, and histopathology of the plaques to determine the potential presence of calcification, and the extent of different types of calcification. The results obtained in users and non-users of warfarin therapy were compared.

## Materials and Methods

This observational study was conducted at Helsinki University Hospital (HUS) in Finland in collaboration with the departments of neurology, vascular surgery, radiology, and pathology. Patients were referred for CEA from the Hospital District of Helsinki and Uusimaa (the total patient population in this district is ~1.5 million) due to a moderate- or high-grade of carotid artery stenosis, and the decision to perform CEA was based in each case on the guidelines of the European Stroke Organization (22). Between October 2012 and September 2015, we recruited 500 consecutive patients who were due to undergo CEA because of advanced atherosclerotic carotid stenosis, either symptomatic (typically following carotid territory neurological symptoms) or asymptomatic (typically following carotid Doppler ultrasound findings after non-specific cerebral symptoms). Patients were recruited consecutively either by a research assistant (S.K.) or a neurologist (P.I., K.N., L.S.). Exclusion criteria were severe aphasia or inability to give informed consent. All consenting eligible CEA patients, except for those undergoing CEA during holiday periods, were recruited. There was no randomization, and no study-related interventions were performed. All patients were interviewed and examined before CEA, except those patients who had emergency surgery, who were interviewed postoperatively. Carotid plaques (CPs) were collected immediately after CEA. Full methodological details about the morphological and histological investigation of CPs in these patients have been published previously (23). The study was approved by the local medical ethics committee and all study patients gave written informed consent. The data that support the findings of this study are available from the corresponding author upon reasonable request.

### Imaging Protocol

**Figure 1:**
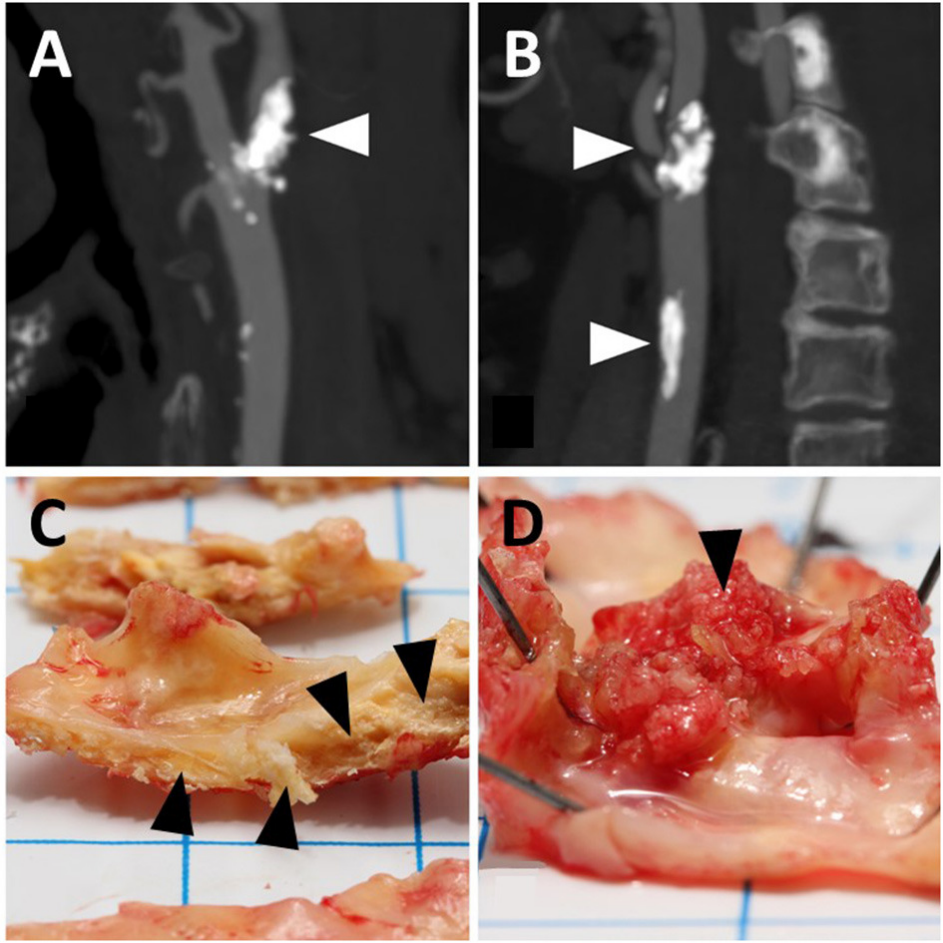
Representative illustrations of calcification in angiograms **(A,B)** and carotid endarterectomy samples for macroscopic observation **(C,D)**. **(A)** Shows calcification of the internal carotid artery (arrowhead); **(B)** shows that calcification is present in the internal (upper arrowhead) as well as the common carotid artery (lower arrowhead). **(C)** shows intramural calcification (arrowheads); **(D)** shows luminal calcification (arrowhead). the angiograms and endarterectomy samples do not refer to the same patients.

Before recruitment, most patients (n = 477) underwent multi-detector carotid artery CTA. If a patient had contraindications for CTA, magnetic resonance angiography (MRA) of the carotid arteries was performed instead. The Meilahti HUS CTA protocol has been described in detail previously (24). Representative CTA images of the internal carotid artery (ICA) and the common carotid artery (CCA) calcification are shown in Figures 1A,B.

Imaging data were analyzed more extensively than required by standard clinicoradiological evaluation guidelines. Carotid CTAs were primarily analyzed at a 3D reformatting station (Advantage Workstation, AW 4.4; GE Medical Systems) by two experienced neuroradiologists (L.V., H.S.) together with a radiology resident (S.M.K.) specifically trained to analyze carotid CTAs; all were blinded to warfarin treatment. Carotid MRAs were excluded from the calcification analysis because of the high specificity of CTA in visualizing calcified carotid artery structures. Thus, our analysis focused on the visual grading of the amount of carotid artery calcification.

### Radiological Classification of Carotid Calcification

The final classification of the carotid CTA calcification was performed using the Impax workstation (AGFA Impax version 6.6.1.5003) from high-resolution calibrated medical monitors. CCA and ICA were visualized from thin axial source images, as well as from sagittal and coronal reformations. The CCA was evaluated throughout its length, i.e., from the aortic arch to the bifurcation level. However, the bifurcation area of the CCA was included in the ICA grading. While the entire length of the CCA was classified for calcification, the classification of ICA calcification was limited to the length encompassed by the typical operative extent of CEA.

CCA calcification (Figure 1B) was graded as Class 0 if no significant calcification was seen along its course (one small spot-like calcification with an estimated diameter of 0.5 mm or less was allowed) and as Class 1 if several small spot-like calcifications or one larger calcification was detected. ICA calcification was graded as Class 0 if the stenosing plaque did not contain any calcification, and thus the domains predominantly contained lipid-like components. As for the CCA, one or two small spot-like calcifications in the plaque area were also allowed with a limit of up to 0.5 mm. The ICA calcification (Figure 1A) was graded as Class 1 if numerous small calcifications were detected in the plaque area; however, the presence of lipid-like components was not excluded from Class 1, even as a dominant feature (heterogeneous plaque structure). In addition, this ICA Class included calcifications observed as ring-like structures, surrounding a lipid-containing stenotic plaque. Fully calcified stenotic plaques with a uniform bulky calculus as the clear dominant plaque feature were graded as Class 2.

### Macroscopic Evaluation of Calcification

The carotid CEA specimens were photographed and macroscopically evaluated based on their visual and morphological characteristics, as previously described (23). The macroscopic evaluations of the calcification revealed that in a fraction of the specimens the entire calcification was located intramurally, i.e., within the vessel wall, while in another fraction of the specimens the calcification had broken the luminal surface of the specimen and extended into the lumen. Accordingly, two forms of CP calcification could be distinguished: purely intramural calcification (Figure 1C) and calcification extending into the lumen referred to as “luminal calcification” (Figure 1D). Regarding the purely intramural location, the calcification was graded into three categories: 0 = no calcified areas, 1 = small calcified areas, and 2 = large heavily calcified areas. The luminal calcifications had broken the surface of the carotid plaque, and they differed macroscopically from the purely intramural calcifications in that they resembled coral reefs, as has also been observed in calcified aortic walls (25). The coral-like projections were graded dichotomously: 0 = no luminal calcification and 1 = luminal calcification.

### Histopathology

**Figure 2:**
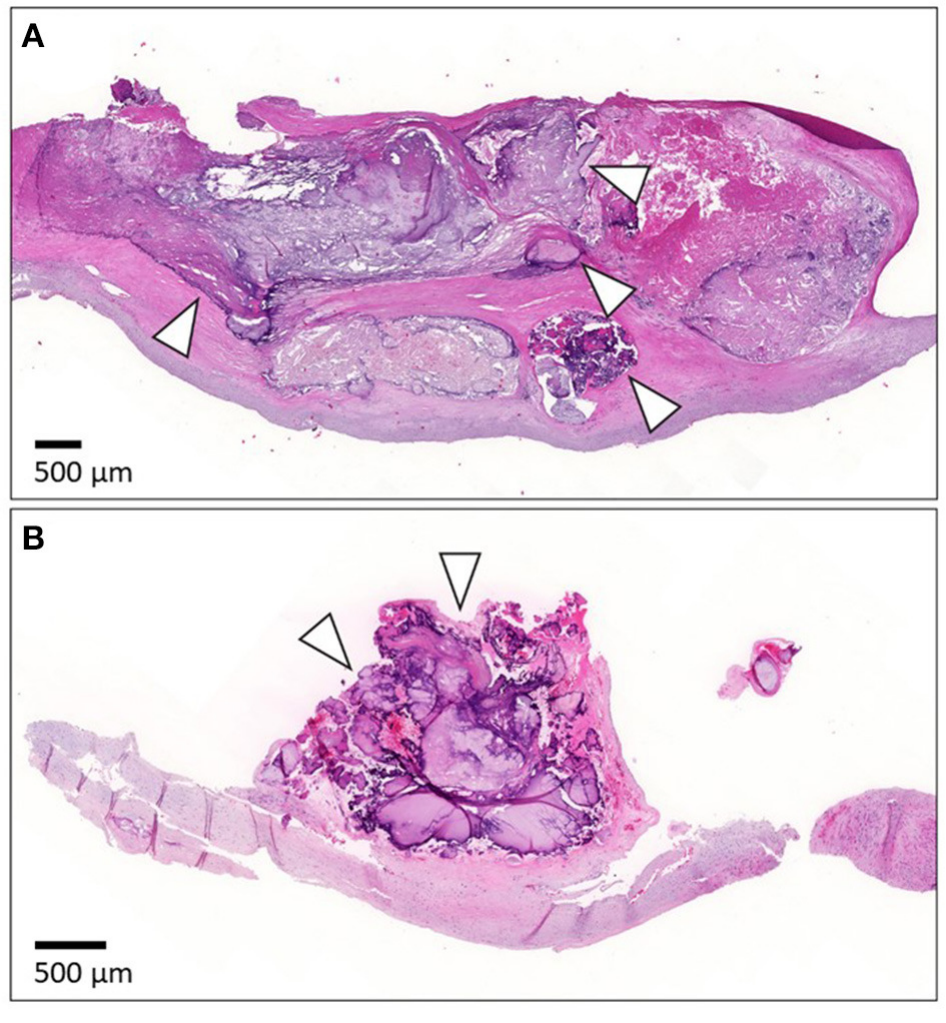
Representative images of calcification in histological samples. **(A)** Shows histology of the intramural calcification and arrowheads point to areas of calcification. **(B)** Presents the histology of luminal calcification (arrowheads). For histology, the specimen was cut longitudinally. The luminal side is upward. The scale bar is 500 μm.

Histopathological evaluation was carried out using one representative longitudinal slice with two histological stains: Hematoxylin and eosin (HE) and Masson's trichrome (MT). Slices were fixed in 10% formalin for 2–4 days, decalcified in EDTA-decalcifying solution for 1–4 weeks depending on the level of calcification, dehydrated, and embedded in paraffin. Sections (4-μm thick) from paraffin-embedded specimens were stained automatically with HE and manually with MT stain. Full details of the methodology have been reported previously (23). Intramural calcification (Figure 2A) was approximated as a percentage of the total plaque area, while luminal calcification (Figure 2B) was quantified as a percentage of the luminal length of the calcification from the total luminal plaque length in the section.

### Statistical Analysis

To determine the significance of the observed effects we formulated multivariable logistic regression models and tested a total of 12 different hypotheses. These models contained different combinations of main effects for gender, age, smoking, hypertension, diabetes, coronary artery disease, renal insufficiency, dyslipidemia, use of statin therapy, and use of warfarin. The response variables were measures of calcification: calcification of either the ICA or CCA in radiological analysis, and intramural and luminal CP calcification in *ex vivo* morphological analysis. Each hypothesis was tested on all these calcification types. The results are reported only for the model containing gender, age, smoking, and warfarin use because the other variables did not reach statistical significance in the tested models (except diabetes for luminal CP calcification, odds ratio [OR] 1.16, *P* = 0.04).

The analysis was complicated by the fact that all warfarin users had radiological calcification of the ICA. This phenomenon is known as “separation.” For datasets that showed separation, the usual maximum likelihood-based estimation of logistic regression models does not allow customary estimation of odds ratios or confidence intervals. Therefore, we estimated the models using Firth's bias-reduced logistic regression (26). Firth's method results in narrower confidence intervals than the traditional logistic regression in a case of complete separation, but they might still be much wider than what one is used to encounter (27).

To determine the significance of the observed histological effects, a linear regression analysis was performed. Histological variables were measured on a percentage scale, which, however, could not be successfully transformed to resemble a normal distribution. Therefore, the significance was evaluated with a permutation test.

All analyses were performed in R 3.3.1 (28). Firth's method was used as implemented in the add-on package logistf (29). A *P* < 0.05 was considered statistically significant, and all reported confidence intervals (CIs) had a 95% coverage. Only complete cases, with no missing observations in the explanatory variables, were used for analyses.

## Results

Of the 500 study patients, 82 had received warfarin due to AF; the median duration of warfarin therapy was 1.6 years. Because the duration of warfarin therapy was not significantly associated with the degree of calcification, a dichotomous parameter describing warfarin therapy was used in the statistical analysis: 0 = the patient had never received warfarin therapy and 1 = the patient had received warfarin therapy for any period of time. Among all patients, 324 had a symptomatic CP, 102 had an asymptomatic CP, and in 74 cases it was uncertain whether the symptomatology was related to the CP. A small number of study patients did not undergo CEA, or their clinical data were missing, and accordingly, 479 patients (and CPs) were included in the multivariable analyses. For some patients, carotid artery MRA was performed instead of CTA; hence, 457 patients were included in the radiological data analyses and histological data were available for 477 CPs. The characteristics of the study patients are presented in Table 1.

**Table 1:** Characteristics of study patients.

	**Warfarin users (*n* = 82)**	**Warfarin non-users (*n* = 418)**	***P*^†^**
Gender (male/female)	60/22	278/140	0.249
Age (median, years)	74.5	69.0	<0.0001^*^
Smoking	10 (12%)	147 (35%)	<0.0001^*^
Symptomatic carotid plaque	31 (38%)	293 (70%)	<0.0001^*^
**Comorbidities**			
Atrial fibrillation	67 (82%)	19 (5%)	<0.0001^*^
Hypertension	64 (78%)	343 (82%)	0.035^*^
Diabetes	30 (37%)	137 (33%)	0.634
Hyperlipidemia	74 (90%)	381 (91%)	0.933
Coronary artery disease	47 (57%)	137 (33%)	0.025^*^
ASO	16 (20%)	70 (17%)	0.430
**Medication**			
DM with medication	26 (32%)	131 (31%)	1.000
Dyslipidemia medication	80 (98%)	395 (95%)	0.403
ATR blocker	30 (37%)	152 (36%)	1.000
ACE inhibitor	29 (35%)	155 (37%)	0.900
Beta blocker	70 (85%)	207 (50%)	<0.0001^*^
Calcium channel blocker	23 (28%)	159 (38%)	0.103

† *Fisher's exact test was used in all comparisons except for age, which was analyzed using t-test*.

*Statin medication in all patients, expect for six who had only ezetimibe; of the patients treated with ezetimibe, one also received warfarin and five were warfarin non-users*.

*ASO, peripheral arterial disease; DM, diabetes mellitus; ATR, angiotensin receptor; ACE, angiotensin converting enzyme*.

* *Indicates statistically significant (p < 0.05)*.

**Figure 3:**
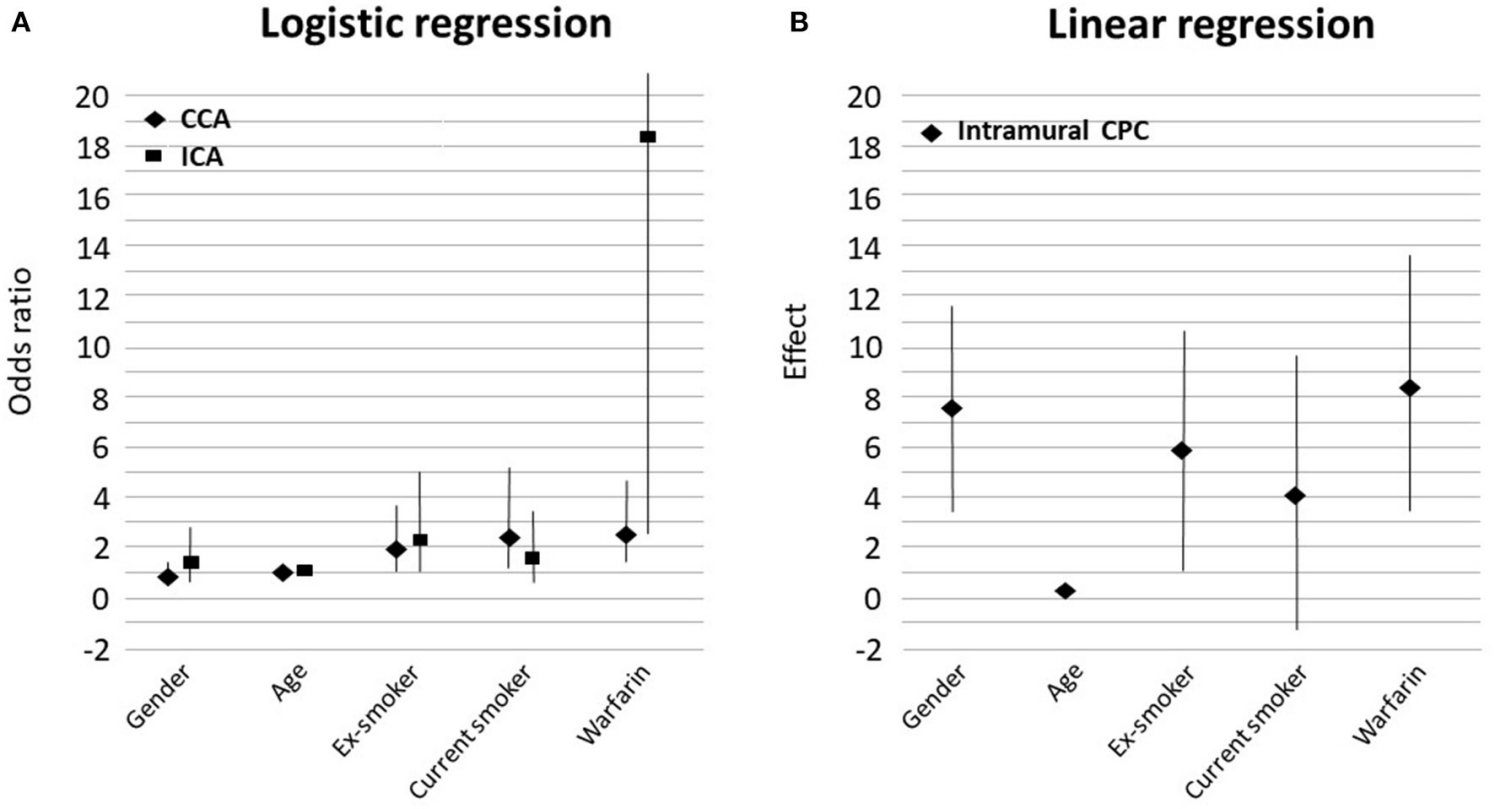
Graphs showing the strength of association between variables in the multivariable model and the radiological evidence of calcification **(A)** and linear regression results for the histological evidence of calcification **(B)**. Female gender favored intramural calcification. CCA, common carotid artery; ICA, internal carotid artery; CPC, carotid plaque calcification.

Warfarin use was associated with increased vascular calcification, as observed in carotid artery CTAs (Figure 3A). Warfarin users had significantly more vascular calcification in their CCAs (OR 2.64, 95% CI 1.51–4.63, *P* = 0.001, Figure 1B) and markedly more in their ICAs (OR 18.27, 95% CI 2.53–2323, *P* < 0.001, Figures 1A, 3A, Table 2). The CCA calcifications were generally small and non-stenosing, whereas those in the ICA were more prominent and associated with the stenosing CP. Coronary artery disease was observed more frequently in warfarin users (57 vs. 33%, *P* = 0.025) than non-users, but hypertension was less common (78 vs. 82%, *P* = 0.035, Table 1). Consistent with the expected high frequency of AF among warfarin users (82 vs. 5%, *P* < 0.001), the use of beta-blocking medication was also more common than in non-users (85 vs. 50%, *P* < 0.001). Warfarin users tended to have tighter carotid stenosis than non-users (degree of stenosis 69.4 vs. 63.7%), but this difference was not statistically significant. Given the nominal effects of warfarin in preventing thromboembolism, it was an expected finding that patients on warfarin had less symptomatic carotid stenosis than non-users (31/82 = 38 vs. 293/418 = 70%, *P* < 0.001). CP calcification, as observed in CTAs, was not associated with the symptom status of the plaque.

**Table 2:** Results of the multivariable logistic regression model for radiological calcification (upper) and macroscopic calcification (lower).

	**CCA**				**ICA^†^**			
**Independent variable**	**OR**	**CI 2.5%**	**CI 97.5%**	***P***	**OR**	**CI 2.5%**	**CI 97.5%**	***P***
(Intercept)	0.0150	0.0014	0.1591	0.0000^*^	0.0366	0.0021	0.4795	0.0105^*^
Gender	0.8208	0.4950	1.3610	0.4440	1.3739	0.7147	2.7583	0.3467
Age	1.0360	1.0048	1.0680	0.0230^*^	1.0650	1.0289	1.1080	0.0002^*^
Ex-smoker	1.9372	1.0484	3.5797	0.0350^*^	2.2587	1.0168	5.0213	0.0455^*^
Current smoker	2.4842	1.2128	5.0887	0.0130^*^	1.5385	0.6888	3.4165	0.2905
Warfarin	2.6425	1.5088	4.6282	0.001^*^	18.2727	2.5307	2323.1240	0.0006^*^
	**Intramural CP calcification**				**Luminal CP calcification**			
**Independent variable**	**OR**	**CI 2.5%**	**CI 97.5%**	***P***	**OR**	**CI 2.5%**	**CI 97.5%**	***P***
(Intercept)	0.0284	0.0027	0.3022	0.0030^*^	0.1027	0.0159	0.6624	0.0170^*^
Gender	1.8804	1.0446	3.3851	0.035^*^	1.6705	1.1032	2.5296	0.0150^*^
Age	1.0596	1.0265	1.0936	0.0000^*^	1.0085	0.9843	1.0333	0.4930
Ex-smoker	1.8252	0.9459	3.5219	0.0730	1.3215	0.8002	2.1823	0.2760
Current smoker	1.2530	0.6287	2.4969	0.5220	1.5124	0.8543	2.6772	0.1560
Warfarin	1.8235	0.7838	4.2428	0.1630	1.6273	0.9704	2.7290	0.0650

*All response variables were categorical: 0 = no calcification, 1 = calcification detected*.

† *Fitted using Firth's bias-reduced logistic regression to compensate for separation. Unusually wide confidence intervals stem from the complete separation present in the data*.

*OR, Odd's ratio; CI, confidence interval; CCA, common carotid artery; CP, carotid plaque; ICA, internal carotid artery*.

* *Indicates statistically significant (p < 0.05)*.

In multivariable analyses (Table 2), age was significantly associated with increased vascular calcification in CCAs (OR 1.04, 95% CI 1.01–1.07, *P* = 0.023, Figure 3A) and in ICAs (OR 1.07, 95% CI 1.03–1.11, *P* < 0.001, Figure 3A). Smoking had a similar effect: Vascular calcification was increased in CCAs among ex-smokers (OR 1.94, 95% CI 1.05–3.58, *P* = 0.035, Figure 3A) and current smokers (OR 2.48, 95% CI 1.21–5.09, *P* = 0.013, Figure 3A). However, the association between smoking and vascular calcification in ICAs was not uniform, as a significant association was seen among ex-smokers (OR 2.26, 95% CI 1.02–5.02, *P* = 0.046, Figure 3A) but not among current smokers. Other risk factors such as hypertension, diabetes, renal insufficiency, and dyslipidemia were not associated with vascular calcification in CCAs or ICAs, nor was statin use (Table 1). All variables that reached statistical significance were included in the multivariable statistical model.

Our core observation of increased CTA-detectable vascular calcification in warfarin users prompted closer macroscopic investigation of *ex vivo* CEA specimens, and examination of whether differential morphological distributions of CP calcification could be found. Although intramural CP calcification was more often observed in warfarin users (Figure 1C) than non-users (93 vs. 81%, Fisher's exact test *P* = 0.009), this difference did not reach statistical significance in multivariable analyses (OR 1.82, 95% CI 0.78–4.24, *P* = 0.163, Table 2). Occasionally, distinct coral reef-like luminal calcifications were also observed in CPs (Figure 1D), as has been found in human aortas (25). In *ex vivo* analysis, the luminal calcifications protruded from the vessel wall like coral reefs and crumbled into small sand-like grains when cut by a scalpel, whereas the intramural CP calcifications resided within the vessel wall in a laminar fashion and were usually very solid and difficult to cut. Warfarin use tended to be associated with luminal CP calcification (43% calcification in warfarin-users vs. 32% in non-users, Fisher's exact test *P* = 0.069), and this tendency persisted in multivariable analysis (OR 1.63, 95% CI 0.97–2.73, *P* = 0.065; Table 2).

In multivariable analyses of macroscopic CP calcifications, female gender was associated with increased intramural calcification (OR 1.88, 95% CI 1.04–3.39, *P* = 0.035) as well as with luminal calcification (OR 1.67, 95% CI 1.10–2.53, *P* = 0.015; Table 2). Age increased intramural calcification (OR 1.06, 95% CI 1.03–1.09, *P* < 0.001) but not luminal calcification. Other risk factors including smoking, hypertension, renal insufficiency, dyslipidemia, and statin use were not statistically significantly associated with either intramural or luminal CP calcification.

In multivariable linear regression analysis of histological staining, the intramural calcified area of the carotid specimen was 8.5% larger in warfarin users compared with non-users; warfarin use was significantly related to intramural CP calcification (effect size 8.46, 95% CI 3.36–13.56, *P* = 0.0018, Figures 2A, 3B, Table 3). In most CEA specimens, intramural calcification was observed within the atherosclerotic plaque, and only occasionally were minor calcifications observed in the medial layer backing the plaque. In addition, calcification was clearly deposited and mature in the atherosclerotic plaques of warfarin users. Females (7.6%, 95% CI 3.51–11.62, *P* = 0.0001) and ex-smokers (5.9%, 95% CI 1.14–10.63%, *P* = 0.0083) had more intramural CP calcifications, while older patients had less intramural CP calcifications (0.25%, 95% CI 0.02–0.48, *P* = 0.0156; Figure 3B). A correlation between warfarin use and luminal CP calcifications was not confirmed by histological analysis (Table 3). The lack of a correlation may reflect incomplete recovery of luminal calcifications, which easily detach during histological sample preparation.

**Table 3:** Results of the multivariable linear regression model for histological calcification.

	**Intramural CP calcification**				**Luminal CP calcification**			
**Independent variable**	**Effect**	**CI 2.5%**	**CI 97.5%**	***P***	**Effect**	**CI 2.5%**	**CI 97.5%**	***P***
(Intercept)	−10.5556	−27.9381	6.8269	0.9999	−3.6407	−17.4078	10.1264	0.9277
Gender	7.5644	3.5092	11.6197	0.0001^*^	0.5453	−2.6665	3.7571	0.3669
Age	0.2516	0.0230	0.4803	0.0156^*^	0.1310	−0.0500	0.3121	0.0694
Ex-smoker	5.8842	1.1381	10.6303	0.0083^*^	−0.4726	−4.2316	3.2863	0.6018
Current smoker	4.1863	−1.2210	9.5936	0.0692	0.0210	−4.2617	4.3036	0.4982
Warfarin	8.4588	3.3606	13.5570	0.0018^*^	1.6758	−2.3621	5.7136	0.2022

* *Indicates statistically significant (p < 0.05)*.

## Discussion

This is the first report on increased carotid plaque calcification in patients on warfarin therapy. The association was strongest for radiologically determined calcification in the ICA (OR 18.27, 95% CI 2.53–2323, *P* < 0.001). The results confirm the previously observed association between calcification and warfarin use in other arterial beds in animal models (10, 11, 16), as well as in humans (12–19). In these human studies, such association was observed in coronary arteries (15, 16), peripheral arteries (medial pattern of calcification) (17), aorta (18), and aortic valve leaflets (19).

The pathology underlying atherosclerotic calcification is still unclear. Multiple sources of calcium have been proposed, including (a) apoptosis of smooth muscle cells (SMCs) or macrophages; (b) release of matrix vesicles, resembling bone formation; (c) diminished inhibition of calcification through deficiency of circulating mineralization inhibitors; and (d) bone generation resulting from perturbed differentiation of vascular SMCs or stem cells (3, 30–35). Furthermore, despite the long-term use of the vitamin K antagonist warfarin (since the 1950s), it took more than half a century to discover that this anticoagulant also affects the mineralization process of both bone and soft tissues (36). In fact, a preventive role for vitamin K in cardiovascular disease has been proposed based on its action as an activator of matrix Gla protein, a calcification inhibitor that is also expressed in vascular tissue (37). We realize that our methodology and findings do not permit elucidation of the mechanistic pathways leading to carotid artery calcification in our cohort. However, the present data revealed an ~20-fold increase in the occurrence of calcification in association with warfarin therapy, which accords with the notion that extended suppression of vitamin K-dependent vascular matrix Gla protein plays an important role in the calcification of atherosclerotic plaques.

The prognostic implications of CP calcification relative to subsequent vascular events have been investigated in several studies and with variable results. Allison and co-workers evaluated computed tomography scans from 4,544 patients and examined the presence of calcium in different arterial beds; the authors observed that, depending on the major artery involved, the calcification had differential prognostic effects. Thus, calcifications in the carotid arteries and in the thoracic aorta showed the most robust association with poor patient survival, the carotid calcification showing statistically significant hazard ratio (HR) values for premature all-cause death which ranged from 1.60 to 1.96 in multivariable models (38). In the Northern Manhattan study, CP calcification of 1,118 stroke-free subjects was assessed using high-resolution B-mode ultrasound. Patients with CP calcification had a significantly increased risk for combined vascular outcome (HR 2.5, 95% CI 1.0–5.8) compared to patients without plaques (39). Of note, Henein et al. found that long-term statin therapy accelerated coronary artery calcification; however, despite this increase in coronary calcification, the number of coronary events did not increase suggesting plaque repair and stabilization during statin treatment (40). In the present study, inclusion of statin use as a variable in the multivariable analysis did not affect the major observation, hence calcification in our study population was not explained by statin use.

In our cohort, the majority (83%) of calcifications represented the intramural type i.e., they were mainly located within the atherosclerotic plaques. Some calcifications showed the gross morphological features of coral-like calcification (luminal calcification), which tended to be overrepresented in warfarin users (*P* = 0.065). The clinical significance of calcification, i.e., its role in determining the vulnerability of an advanced atherosclerotic plaque, is still under investigation (4, 35, 41). Generally, calcium in atherosclerotic plaques has been linked with stability (42, 43), but evidence suggests that microcalcifications (typically 10 μm in diameter) derived from dying macrophages or SMCs in thin fibrous caps may trigger plaque rupture through locally increased stress, caused by the mismatch in material properties between the microcalcifications and the fibrous tissue present in plaques (41, 44–46). The different macroscopic and histological appearances of the calcification may represent different pathophysiological processes, in line with suggestions by other investigators (47), and may have different prognostic value. We realize that the results of each of the calcification grading method used in the present work yielded somewhat arbitrary results. However, all three different gradings complemented each other and provided as multifaceted picture of the phenotypes of the calcification process.

It could be argued that patients on warfarin had more advanced atherosclerotic disease in general and hence also more calcification in their carotid arteries, i.e., the association observed could be confounded by the indication. Indeed, there was a slight overrepresentation of coronary artery disease (Table 1) among warfarin users in this study. Importantly, our results are in line with previous findings by Peeters et al. who found that vascular (aortic) calcification was increased in AF patients who used a vitamin K antagonist (warfarin), and that such association was not present in AF patients taking one of the new direct oral anticoagulants that are non-vitamin K antagonists (18).

It has been acknowledged, however, that although atherosclerosis is a systemic disease, its burden is not similar across different vessel beds (38, 48). Moderate to strong correlations have been found between calcification in coronary arteries, aortic arch, and carotid arteries, implying that assessment of the amount of calcification in one arterial bed only gives a rough estimate of calcification in other atherosclerosis-susceptible arterial segments (49). Although all subjects in our study undergoing CEA had variable manifestations of long-standing general atherosclerotic disease and advanced stenosing carotid artery plaques, no significant difference in the degree of carotid stenosis was found between warfarin users and non-users. Since the degree of stenosis roughly reflects plaque size and therefore also disease stage, the above finding suggests that calcification reflected differences in the biochemical and pathophysiological processes in plaques derived from different patients rather than merely the stage of the disease.

The association between warfarin use and increased vascular calcification is a concern because of the wide use of warfarin, especially in patients with atherosclerotic cardiovascular disease, and the positive association between arterial calcification and vascular events and all-cause mortality (38). Our data do not establish whether calcification should be considered a marker of a vulnerable atherosclerotic plaque or rather a sign of plaque evolution toward “end-stage” stabilization and hence a better prognosis. We observed that although the CPs were more calcified in warfarin users than in non-users, in the user group there were fewer symptomatic plaques (38 vs. 70%, *P* < 0.0001). This finding may imply that arterial calcification signifies *in situ* plaque stabilization, at least in the carotid arterial segment studied here. However, during anticoagulation, the thromboembolic event originating from the plaque may have been prevented, which may have at least partially concealed the underlying atherosclerotic vasculopathic process and its prothrombotic and thromboembolic potential.

Finally, the clinical significance of our findings and the potential additional impact of accelerated calcification on the evolution of carotid disease remain to be determined. However, the results of this study reveal that warfarin use is associated with calcification of carotid atherosclerotic plaques present in the bifurcation area, i.e., in the predilection site of stroke-causing plaques. Further investigations may also address the evolution of carotid calcification in patients on anticoagulants lacking an effect on vitamin K.

## Data Availability Statement

The original contributions presented in the study are included in the article/supplementary material, further inquiries can be directed to the corresponding authors.

## Ethics Statement

The studies involving human participants were reviewed and approved by HUS eettinen toimikunta, PL 705, 00029 HUS Biomedicum Helsinki 2 C 7.krs, Tukholmankatu 8 C, Helsinki. The patients/participants provided their written informed consent to participate in this study.

## Author Contributions

KN: design, data analysis, drafted the manuscript for intellectual content, data acquisition, and interpretation of the data. SMK: design, data acquisition, analyzed the data (radiology), and manuscript revision. LM: design, data acquisition, histological analysis, and manuscript revision. JT: statistical analysis of the data. PI: design, data acquisition, interpreted the data, and manuscript revision. HH, JS, and PS: design, data acquisition, and manuscript revision. PV: responsible for the carotid endarterectomies and manuscript revision. SK: data acquisition (patient interviews, coordination). IP: design and conceptualized study on pharmacologic viewpoint and manuscript revision. HS and LV: design and conceptualized study on radiologic viewpoint and manuscript revision. MM: design, interpreted the data (histology), and manuscript revision. LS: design, interpreted the data, and manuscript revision. PK and PL: design and conceptualized study, interpreted the data, and manuscript revision. All authors contributed to the article and approved the submitted version.

## Conflict of Interest

JT received a lecture fee from Boehringer-Ingelheim in 2014. MM has received lecture fees from Boehringer-Ingelheim, Bristol-Myers Squibb, MSD, and Takeda, as well as congress sponsorship and educational travel support from Pfizer, Roche, Bristol-Myers Squibb, and MSD. PK has received consultancy or lecture fees from Amgen, Novartis, Raisio Group, and Sanofi. The remaining authors declare that the research was conducted in the absence of any commercial or financial relationships that could be construed as a potential conflict of interest.

## References

[1] Bäck M Yurdagul AJ Tabas I Öörni K Kovanen PT. Inflammation and its resolution in atherosclerosis: mediators and therapeutic opportunities. Nat Rev Cardiol. (2019) 16:389–406. 10.1038/s41569-019-0169-2 30846875 PMC6727648

[2] Tajbakhsh A Kovanen PT Rezaee M Banach M Sahebkar A. Ca^2+^ Flux: searching for a role in efferocytosis of apoptotic cells in atherosclerosis. J Clin Med. (2019) 8:2045. 10.3390/jcm8122047 31766552 PMC6947386

[3] Thompson B Towler DA. Arterial calcification and bone physiology: role of the bone-vascular axis. Nat Rev Endocrinol. (2012) 8:529–43. 10.1038/nrendo.2012.36 22473330 PMC3423589

[4] Alexopoulos N Raggi P. Calcification in atherosclerosis. Nat Rev Cardiol. (2009) 6:681–8. 10.1038/nrcardio.2009.165 19786983

[5] Virmani R Burke AP Kolodgie FD Farb A. Vulnerable plaque: the pathology of unstable coronary lesions. J Intervent Cardiol. (2002) 15:439–46. 10.1111/j.1540-8183.2002.tb01087.x 12476646

[6] Savelieva I John Camm A. Atrial fibrillation and heart failure: natural history and pharmacological treatment. Europace. (2004) 5(Suppl 1):S5–19. 10.1016/j.eupc.2004.07.003 15450275

[7] Aguilar MI Hart R. Oral anticoagulants for preventing stroke in patients with non-valvular atrial fibrillation and no previous history of stroke or transient ischemic attacks. Cochrane Database Syst Rev. (2005) 3:CD001927. 10.1002/14651858.CD001927.pub2 16034869 PMC8408914

[8] January CT Wann LS Alpert JS Calkins H Cigarroa JE Cleveland JC. 2014 AHA/ACC/HRS guideline for the management of patients with atrial fibrillation: a report of the American College of Cardiology/American Heart Association Task Force on Practice Guidelines and the Heart Rhythm Society. J Am Coll Cardiol. (2014) 64:e1–76. 10.1016/j.jacc.2014.03.022 24685669

[9] Kirchhof P Benussi S Kotecha D Ahlsson A Atar D Casadei B. 2016 ESC Guidelines for the management of atrial fibrillation developed in collaboration with EACTS. Eur Heart J. (2016) 37:2893–2962. 10.1093/eurheartj/ehw210 27567408

[10] Howe AM Webster WS. Warfarin exposure and calcification of the arterial system in the rat. Int J Exp Pathol. (2000) 81:51–6. 10.1046/j.1365-2613.2000.00140.x 10718864 PMC2517807

[11] Price PA Faus SA Williamson MK. Warfarin causes rapid calcification of the elastic lamellae in rat arteries and heart valves. Arterioscler Thromb Vasc Biol. (1998) 18:1400–7. 10.1161/01.ATV.18.9.1400 9743228

[12] Tantisattamo E Han KH O'Neill WC. Increased vascular calcification in patients receiving warfarin. Arterioscler Thromb Vasc Biol. (2015) 35:237–42. 10.1161/ATVBAHA.114.304392 25324574

[13] Schori TR Stungis GE. Long-term warfarin treatment may induce arterial calcification in humans: case report. Clin Invest Med. (2004) 27:107–9. 15202830

[14] Fusaro M Tripepi G Noale M Plebani M Zaninotto M Piccoli A. Prevalence of vertebral fractures, vascular calcifications, and mortality in warfarin treated hemodialysis patients. Curr Vasc Pharmacol. (2015) 13:248–58. 10.2174/15701611113119990146 23927679

[15] Weijs B Blaauw Y Rennenberg RJ Schurgers LJ Timmermans CC Pison L. Patients using vitamin K antagonists show increased levels of coronary calcification: an observational study in low-risk atrial fibrillation patients. Eur Heart J. (2011) 32:2555–62. 10.1093/eurheartj/ehr226 21775389

[16] Schurgers LJ Joosen IA Laufer EM Chatrou ML Herfs M Winkens MH. Vitamin K-antagonists accelerate atherosclerotic calcification and induce a vulnerable plaque phenotype. PLoS ONE. (2012) 7:e43229. 10.1371/journal.pone.0043229 22952653 PMC3430691

[17] Han KH O'Neill WC. Increased peripheral arterial calcification in patients receiving Warfarin. JAHA. (2016) 5:e002665. 10.1161/JAHA.115.002665 26811161 PMC4859382

[18] Peeters FE Dudink EA Kimenai DM Weijs B Altintas S Heckman LI. Vitamin K antagonists, non-vitamin k antagonist oral anticoagulants, and vascular calcification in patients with atrial fibrillation. TH Open. (2018) 2:e391–8. 10.1055/s-0038-1675578 31249966 PMC6524908

[19] Yamamoto K Koretsune Y Akasaka T Kisanuki A Ohte N Takenaka T. Effects of vitamin K antagonist on aortic valve degeneration in non-valvular atrial fibrillation patients: prospective 4-year observational study. Thromb Res. (2017) 160:69–75. 10.1016/j.thromres.2017.10.027 29121522

[20] Byington RP Evans GW Espeland MA Applegate WB Hunninghake DB Probstfield J. Effects of lovastatin and warfarin on early carotid atherosclerosis: sex-specific analyses. Asymptomatic Carotid Artery Progression Study. (ACAPS) Research Group. Circulation. (1999) 100:e14–7. 10.1161/01.cir.100.3.e14 10411862

[21] Peeters MTJ Houben R Postma AA van Oostenbrugge RJ Schurgers LJ Staals J. Vitamin K antagonist use and risk for intracranial carotid artery calcification in patients with intracerebral hemorrhage. Front Neurol. (2019) 10:1278. 10.3389/fneur.2019.01278 31920910 PMC6933022

[22] European Stroke Organisation (ESO) Executive Committee. ESO Writing Committee. Guidelines for management of ischaemic stroke and transient ischaemic attack 2008. Cerebrovasc Dis. (2008) 25:457–507. 10.1159/000131083 18477843

[23] Nuotio K Ijäs P Heikkilä HM Koskinen SM Saksi J Vikatmaa P. Morphology and histology of silent and symptom-causing atherosclerotic carotid plaques-Rationale and design of the Helsinki Carotid Endarterectomy Study 2 (the HeCES2). Ann Med. (2018) 50:501–10. 10.1080/07853890.2018.1494851 30010425

[24] Koskinen SM Soinne L Valanne L Silvennoinen H. The normal internal carotid artery: a computed tomography angiographic study. Neuroradiology. (2014) 56:723–9. 10.1007/s00234-014-1394-3 24969944

[25] Schlieper G Grotemeyer D Aretz A Leon JS Krüger T Rehbein H. Analysis of calcifications in patients with coral reef aorta. Ann Vasc Surg. (2010) 24:408–14. 10.1016/j.avsg.2009.11.006 20144533

[26] Firth D. Bias reduction of maximum likelihood estimates. Biometrika. (1993) 80:27–38. 10.1093/biomet/80.1.27

[27] Mansournia M Geroldinger A Greenland S Heinze G. Separation in logistic regression: causes, consequences, and control. Am J Epidemiol. (2018) 187:864–70. 10.1093/aje/kwx299 29020135

[28] R Development Core Team. R: A Language and Environment for Statistical Computing (2016). Available online at: https://www.R-project.org/

[29] Heinze G Ploner M. Firth's Bias-Reduced Logistic Regression. R package version 1.22 (2016). Available online at: https://CRAN.R-project.org/package=logistf

[30] Villa-Bellosta R. New insights into endogenous mechanisms of protection against arterial calcification. Atherosclerosis. (2020) 306:68–74. 10.1016/j.atherosclerosis.2020.03.007 32209233

[31] Andrews J Psaltis PJ Bartolo BA Nicholls SJ Puri R. Coronary arterial calcification: a review of mechanisms, promoters and imaging. Trends Cardiovasc Med. (2018) 28:491–501. 10.1016/j.tcm.2018.04.007 29753636

[32] Zazzeroni L Faggioli G Pasquinelli G. Mechanisms of arterial calcification: the role of matrix vesicles. Eur J Vasc Endovasc Surg. (2018) 55:425–32. 10.1016/j.ejvs.2017.12.009 29371036

[33] Nakahara T Dweck MR Narula N Pisapia D Narula J Strauss HW. Coronary artery calcification: from mechanism to molecular imaging. JACC Cardiovasc.Imaging. (2017) 10:582–93. 10.1016/j.jcmg.2017.03.005 28473100

[34] Speer MY Giachelli CM. Regulation of cardiovascular calcification. Cardiovasc Pathol. (2004) 13:63–70. 10.1016/S1054-8807(03)00130-3 15033154

[35] Yahagi K Kolodgie FD Lutter C Mori H Romero ME Finn AV. Pathology of human coronary and carotid artery atherosclerosis and vascular calcification in diabetes mellitus. Arterioscler Thromb Vasc Biol. (2017) 37:191–204. 10.1161/ATVBAHA.116.306256 27908890 PMC5269516

[36] Berenjian A Mahanama R Kavanagh J Dehghani F. Vitamin K series: current status and future prospects. Crit Rev Biotechnol. (2015) 35:199–208. 10.3109/07388551.2013.832142 24044610

[37] Shea MK Holden RM. Vitamin K status and vascular calcification: evidence from observational and clinical studies. Adv Nutr. (2012) 3:158–65. 10.3945/an.111.001644 22516723 PMC3648716

[38] Allison MA Hsi S Wassel CL Morgan C Ix JH Wright CM. Calcified atherosclerosis in different vascular beds and the risk of mortality. Arterioscler Thromb Vasc Biol. (2012) 32:140–6. 10.1161/ATVBAHA.111.235234 22034514

[39] Prabhakaran S Singh R Zhou X Ramas R Sacco RL Rundek T. Presence of calcified carotid plaque predicts vascular events: the Northern Manhattan Study. Atherosclerosis. (2007) 195:e197–201. 10.1016/j.atherosclerosis.2007.03.044 17482197 PMC6286814

[40] Henein M Granasen G Wiklund U Schmermund A Guerci A Erbel R. High dose and long-term statin therapy accelerate coronary artery calcification. Int J Cardiol. (2015) 184:581–6. 10.1016/j.ijcard.2015.02.072 25769003

[41] Mori H Torii S Kutyna M Sakamoto A Finn AV Virmani R. Coronary artery calcification and its progression: what does it really mean? JACC Cardiovasc.Imaging. (2018) 11:127–42. 10.1016/j.jcmg.2017.10.012 29301708

[42] Finn AV Nakano M Narula J Kolodgie FD Virmani R. Concept of vulnerable/unstable plaque. Arterioscler Thromb Vasc Biol. (2010) 30:1282–92. 10.1161/ATVBAHA.108.179739 20554950

[43] Virmani R Burke AP Farb A Kolodgie FD. Pathology of the vulnerable plaque. J Am Coll Cardiol. (2006) 47(Suppl 8):13–18. 10.1016/j.jacc.2005.10.065 16631505

[44] Ladich E Yahagi K Romero ME Virmani R. Vascular diseases: aortitis, aortic aneurysms, and vascular calcification. Cardiovasc Pathol. (2016) 25:432–41. 10.1016/j.carpath.2016.07.002 27526100

[45] Vengrenyuk Y Carlier S Xanthos S Cardoso L Ganatos P Virmani R. A hypothesis for vulnerable plaque rupture due to stress-induced debonding around cellular microcalcifications in thin fibrous caps. Proc Natl Acad Sci USA. (2006) 103:14678–83. 10.1073/pnas.0606310103 17003118 PMC1595411

[46] Cardoso L Weinbaum S. Microcalcifications, Their genesis, growth, and biomechanical stability in fibrous cap rupture. Adv Exp Med Biol. (2018) 1097:129–55. 10.1007/978-3-319-96445-4_7 30315543

[47] Lin R Chen S Liu G Xue Y Zhao X. Association between carotid atherosclerotic plaque calcification and intraplaque hemorrhage: a magnetic resonance imaging study. Arterioscler Thromb Vasc Biol. (2017) 37:1228–33. 10.1161/ATVBAHA.116.308360 28450297

[48] Odink AE van der Lugt A Hofman A Hunink MG Breteler MM Krestin GP. Association between calcification in the coronary arteries, aortic arch and carotid arteries: the Rotterdam study. Atherosclerosis. (2007) 193:408–13. 10.1016/j.atherosclerosis.2006.07.007 16919637

[49] Jashari F Ibrahimi P Nicoll R Bajraktari G Wester P Henein MY. Coronary and carotid atherosclerosis: similarities and differences. Atherosclerosis. (2013) 227:193–200. 10.1016/j.atherosclerosis.2012.11.008 23218802

